# The evolving landscape and research trend of calcitonin gene-related peptide in migraine: A bibliometric analysis and visualization

**DOI:** 10.3389/fneur.2024.1415760

**Published:** 2024-06-24

**Authors:** Liwei Wang, Qing Wang, Huaqiong Diao, Xueying Liu, Yonglie Zhao

**Affiliations:** ^1^Department of Neurology, Beijing University of Chinese Medicine Third Affiliated Hospital, Beijing, China; ^2^Department of Traditional Chinese Medicine, The Sixth Medical Center of PLA General Hospital, Beijing, China

**Keywords:** migraine, CGRP, bibliometric analysis, visualization, CiteSpace, VOSviewer

## Abstract

**Background:**

Migraine is a global public health concern, affecting both social and individual well-being. Calcitonin gene-related peptide (CGRP), a crucial neuropeptide, holds important research value in understanding migraine pathogenesis. CGRP receptor antagonists and monoclonal antibodies that target CGRP or its receptors have shown efficacy in reducing migraine frequency and severity, presenting a promising therapeutic approach. This study aimed to conduct a comprehensive bibliometric analysis to analyze the current state, research trends, and future directions of CGRP in migraine.

**Methods:**

Bibliometric tools including CiteSpace, VOSviewer, etc., were utilized to extract and summarize publications related to CGRP in migraine from the Web of Science Core Collection Database (WOSCC) between 2004 and 2023, as of December 31, 2023. The analysis focused on trends in annual publications, leading countries/regions and institutions, prominent journals and references, influential authors, and high-frequency keywords in the field.

**Results:**

A total of 1,821 articles and reviews involving 5,180 authors from 1,315 organizations across 64 countries were included in the study. These publications were distributed across 362 journals and accumulated 56,999 citations by December 31, 2023. An increasing trend was observed in annual publications on CGRP in migraine. The United States emerged as the leading nation in both publications and citations, with academic Peter Goadsby contributing the highest number of publications. The University of Copenhagen stood out as the institution with the most publications, and *Cephalalgia* emerged as the most influential journal. The most cited paper identified was “Calcitonin gene-related peptide receptor antagonist BIBN4096BS for the acute treatment of migraine” by Jes Olesen, published in the *New Engl Med*. Keyword frequency analysis revealed prevalent terms such as “migraine,” “CGRP,” and “episodic migraine,” along with emerging topics represented by keywords including “trial,” “monoclonal antibodies,” “preventive treatment,” and “safety.”

**Conclusion:**

CGRP is pivotal in migraine pathogenesis, and there is a robust research foundation exploring its role. The US leads in research output on CGRP in migraine. Investigating the mechanism of CGRP and its receptor in migraine remains a key area of interest, particularly focusing on signaling pathways. Future research should target identifying critical therapeutic targets in CGRP antagonist pathways for migraine treatment.

## Introduction

Migraine, a disabling neurological disorder characterized by sensory sensitivity, manifests as unilateral, severe, throbbing headaches accompanied by photophobia, phonophobia, nausea, and vomiting (1–3). It has two major types: migraine without aura and migraine with aura. Migraine without aura is characterized by recurrent headache disorder lasting 4–72 h, often aggravated by routine physical activity. Migraine with aura includes transient focal neurological symptoms that last several minutes, preceding or accompanying the headache, with symptoms like unilateral, fully reversible visual, sensory, or other central nervous system disturbances. Additionally, migraines can be classified as episodic or chronic based on the frequency of attacks. Chronic migraine is defined as experiencing headaches on 15 or more days per month for over 3 months, with migraine features on at least eight of those days. This condition often develops from episodic migraine, which is characterized by prolonged and recurrent episodes. Key factors that increase the risk of progressing from episodic to chronic migraine include the overuse of acute migraine medications, ineffective acute treatments, obesity, and depression (4–6). This complex condition imposes a significant individual burden and is associated with substantial pain interference, often increasing susceptibility to psychiatric comorbidities such as depression within the migraine population (7). Over recent years, the incidence of migraine has been rising among individuals aged 15–49, contributing to a notable increase in both direct and indirect medical costs and imposing a considerable economic burden on society (8, 9).

Calcitonin gene-related peptide (CGRP) is a neuropeptide widely distributed in nociceptive sensory afferent fibers originating from the trigeminal nerve and has the ability to cause nociception, vasodilation, and neurogenic inflammation (10, 11). Scientific studies suggest that the origin of migraine may be vasodilation and aseptic inflammation of the dura mater and involving activation and sensitization of the neurovascular pathways, followed by the involvement of the trigeminovascular system (TVS) (12–15). Upon TVS activation, vasoactive peptides such as CGRP are released in the meninges, causing dural neurogenic inflammation and central sensitization (16–18). During migraine attacks, CGRP induces vasodilation, neurogenic inflammation, and synthesis processes in central events, which contribute to migraine development (10, 19). Studies have shown that CGRP levels increase in the cerebrospinal fluid (CSF), external jugular vein, and other body fluids of migraine patients during migraine attacks (10, 20, 21). As a result, CGRP serves as a potential biological marker to assist in the diagnosis of migraine (22–24). In recent years, the advent of CGRP receptor antagonists (gepants) and anti-CGRP monoclonal antibodies has revolutionized migraine therapy, providing new avenues for both acute and preventive treatments (25). CGRP receptor antagonists (gepants) and anti-CGRP monoclonal antibodies both target the CGRP pathway but differ in mechanisms and applications. Gepants, such as ubrogepant, rimegepant, zavegepant, and atogepant, work by blocking CGRP receptors to inhibit vasodilation and inflammation. This mechanism provides rapid relief with fewer gastrointestinal side effects and a reduced risk of medication overuse headaches compared to traditional therapies like triptans and ergot alkaloids (26, 27). Ubrogepant and zavegepant are primarily used for acute migraine treatment, while atogepant is used for preventive treatment. Rimegepant is notable for being approved for both acute and prophylactic treatment of migraines. The recommended dosage for acute treatment is 75 mg once daily, whereas for prevention, it is administered every other day (28). In contrast, anti-CGRP monoclonal antibodies bind directly to CGRP or its receptor, preventing it from inducing migraine and acting as a prophylactic treatment. These antibodies, administered via injection, have a longer half-life, allowing for less frequent dosing and better patient adherence, making them more suitable for long-term preventive treatment (11). Therefore, the discovery of CGRP and its receptors, alongside the development of anti-CGRP drugs, has provided novel insights into migraine pathophysiology and treatment. What’s more, CGRP-targeted monoclonal antibodies and antagonists are likely to the backbone for the treatment of migraine in the future.

Bibliometric analysis is an effective and scientific method used to assess the status and developmental trends within a specific research field (29). While previous researchers have conducted bibliometric studies on migraine (30, 31), there is a noticeable absence of bibliometric studies specifically focused on CGRP in migraine. This gap hinders researchers from thoroughly understanding the current state of research, international collaboration, and emerging trends in this field. In this study, we employed bibliometric tools such as CiteSpace, VoSviewer, and the R package “bibliometrix” to conduct an in-depth analysis of publications related to CGRP in migraine research (32–34). These tools allowed us to gather detailed information on countries, institutions, journals, references, authors, and keywords within the field and visualize the results of our analysis. To address this knowledge gap, the purpose of this study is to conduct a comprehensive review of CGRP-related publications in migraine research from 2004 to 2023 and to evaluate research hotspots and emerging trends in this field. These findings aim to provide a foundation for enhancing research quality and to contribute to advancements in clinical practices, ultimately benefiting patients with migraine.

## Methods

### Data source and collection

Published papers were systematically retrieved from the Science Citation Index Expanded (SCI-Expanded) of the Web of Science Core Collection (WoSCC) database provided by Clarivate Analytics, covering the period from January 2004 to December 2023. The main inclusion criteria for this study were original articles and reviews focusing on migraine and CGRP, including preclinical and clinical evidence. Exclusion criteria included other literature types such as book chapters and non-English language articles. Articles solely focusing on either migraine or CGRP were also excluded.

### Search strategy

The search strategy employed was as follows (TS = topic search): TS = (“Calcitonin Gene Related Peptide”) OR TS = (“Gene-Related Peptide, Calcitonin”) OR TS = (“Calcitonin Gene-Related Peptide II”) OR TS = (“Calcitonin Gene Related Peptide II”) OR TS = (“beta-CGRP”) OR TS = (“beta-Calcitonin Gene-Related Peptide”) OR TS = (“beta Calcitonin Gene Related Peptide”) OR TS = (“alpha-CGRP”) OR TS = (“alpha-Calcitonin Gene-Related Peptide”) OR TS = (“alpha Calcitonin Gene Related Peptide”) OR TS = (“Calcitonin Gene-Related Peptide I”) OR TS = (“Calcitonin Gene Related Peptide I”) AND TS = (Migraine).

Given the substantial advancements in CGRP research for migraine, the study period covers January 1, 2004, to December 31, 2023, spanning 20 years. Full records of publications and their citations were directly downloaded from the database. Additionally, references cited in relevant studies were hand-searched to identify potentially relevant articles. All retrieved documents were downloaded on December 31, 2023. Two researchers independently screened titles and abstracts, with discrepancies resolved through consultation with a third researcher to reach a final decision.

### Data visualization and analytical methods

The R package “bibliometrix” (version 4.2.3) and Microsoft Excel 2019 were used to preliminary analyze and manage data for bibliometric analysis. CiteSpace 6.2.R4 and VOSviewer 1.6.19 were adopted to generate visualization analysis, characteristic mapping, and optimize diagrams. Cooperation maps of countries/regions and chord diagrams were made by SCImago Graphica 1.0.34. Histograms were made by Origin V9.8. In addition, the latest H-index, SCImago Institutions and Journal Rank, and Impact Factor have been added for a clear and integrated analysis.

## Results

**Figure 1:**
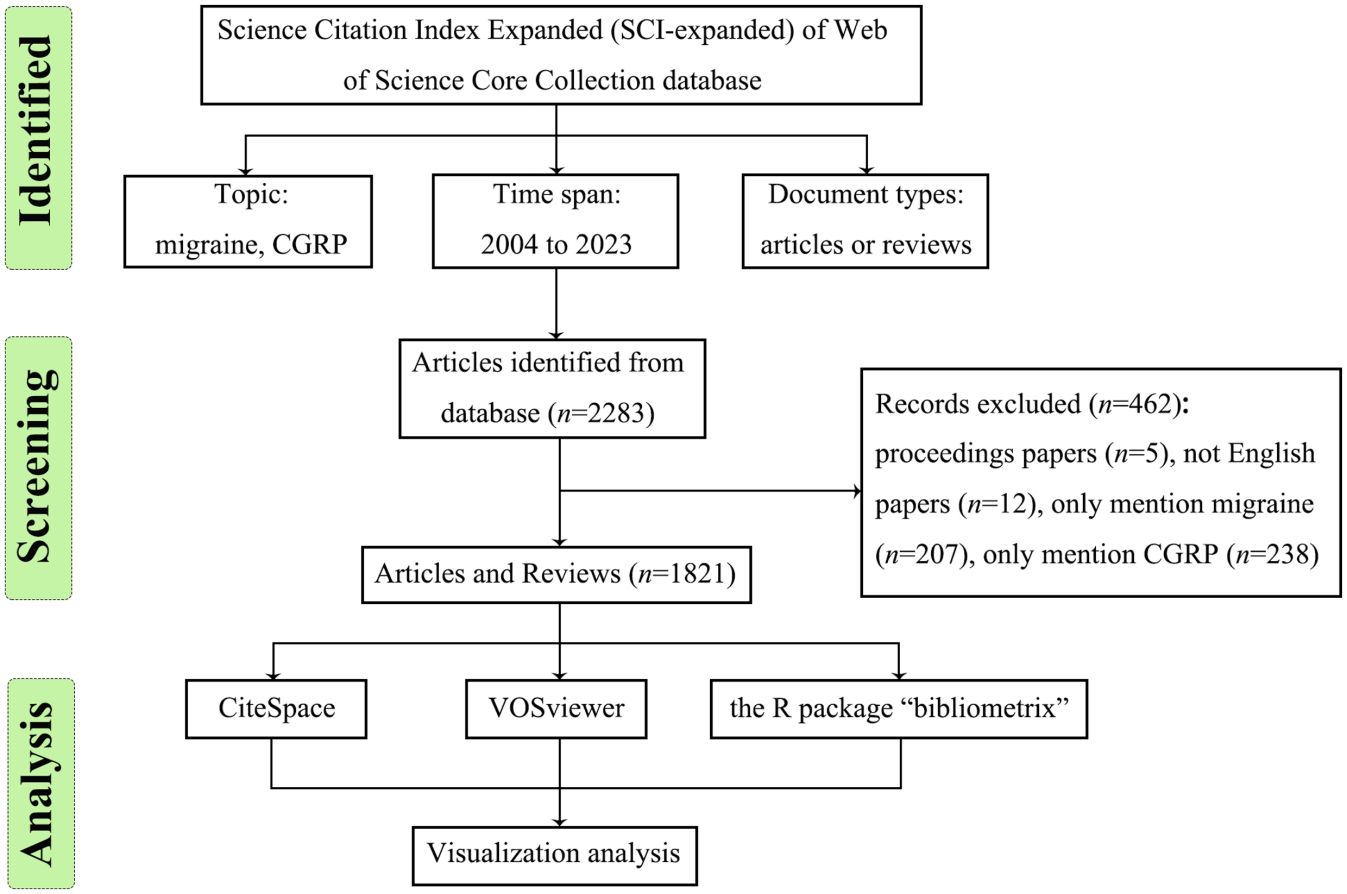
Flowchart of literature selection.

We initially retrieved 2,283 papers using the search strategy in the WoS database. Following the exclusion of conference papers and non-English language articles, we obtained 2,266 records. Furthermore, we manually excluded 445 papers that solely focused on migraine or CGRP, leaving 1,821 papers eligible for bibliometric analysis. Figure 1 illustrates the retrieval flowchart.

### Analysis of publication outputs

**Figure 2:**
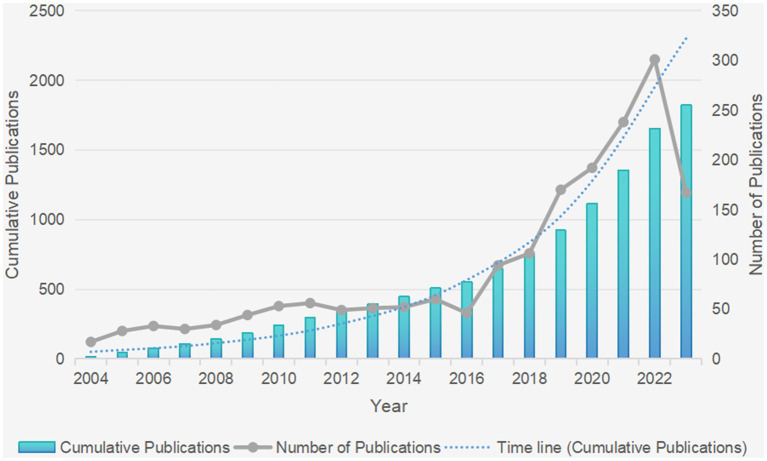
The changing trend of annual publications in CGRP-related research in migraine from 2004 to 2023 (the blue column represents the annual cumulative publications, while the gray line shows the annual number of publications).

The annual output of publications is a crucial indicator of scientific research development, reflecting the evolving trends and degree of attention in a specific field. Figure 2 illustrates a notable upward trajectory in global publications focusing on CGRP in migraine. This growth can be delineated into two distinct phases: an initial period characterized by fluctuating growth from 2004 to 2016, followed by a pronounced acceleration in publications from 2017 to 2022. During the period from 2004 to 2016, the average annual publication output was 42.5 papers. Subsequently, from 2017 to 2022, this figure surged to an average of 183.5 papers per year, indicating a substantial increase in research output. The calculated growth rates for these periods are noteworthy: a growth rate of 7.97% from 2004 to 2016, with publication numbers rising from 17 in 2004 to 46 in 2016; and a more pronounced growth rate of 21.45% from 2017 to 2022, where publications escalated from 94 in 2017 to 301 in 2022 (35). This analysis highlights a promising trend of growth in research within the field of CGRP and migraine. These trends indicate persistent interest and investment in this area of study, which positions it for further development and exploration in the future.

### Analysis of countries/regions and organizations

**Figure 3:**
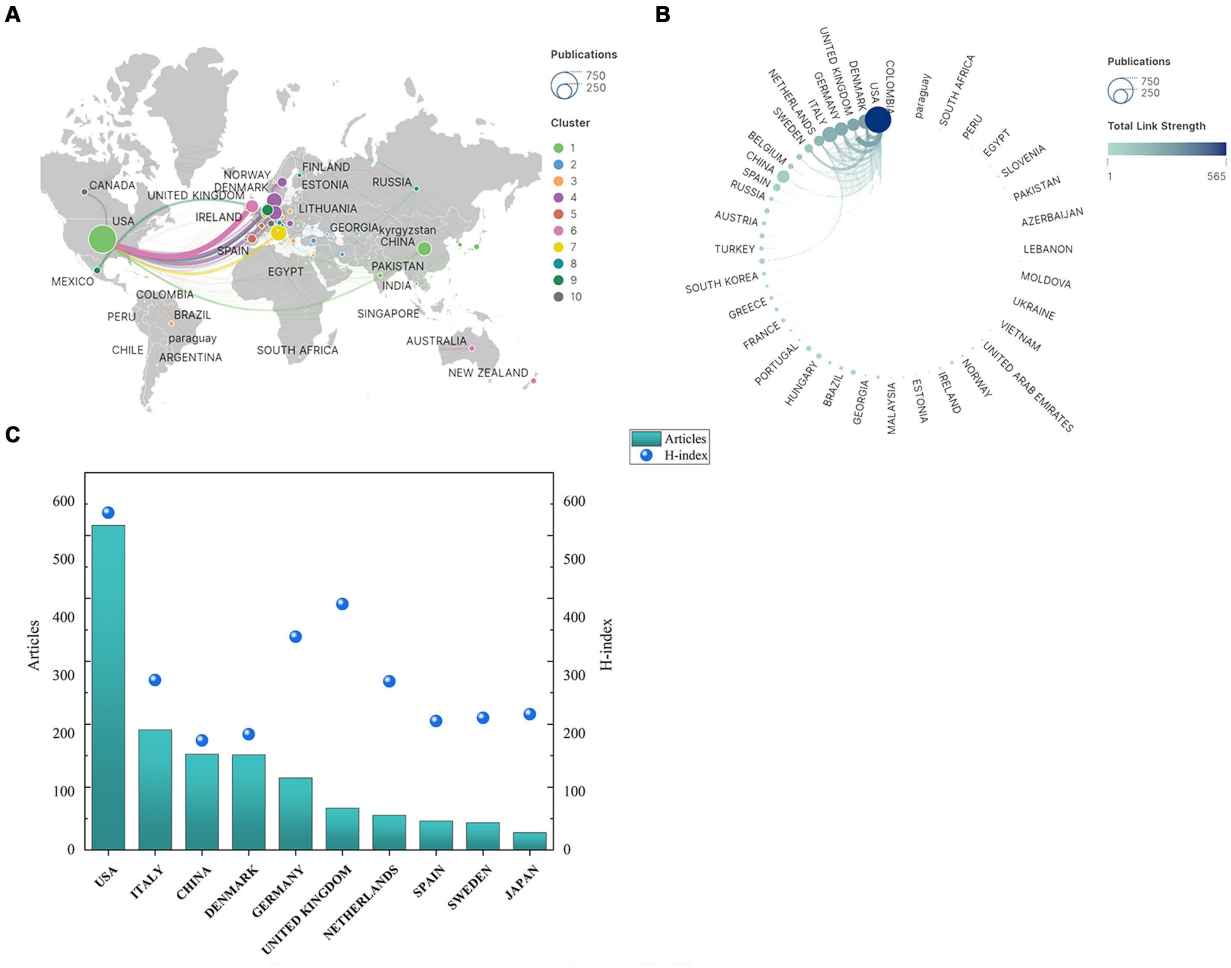
Geographical distribution and cooperation map of countries/regions in CGRP-related research in migraine: **(A)** inter-country cooperation network map; **(B)** inter-country cooperation chord map; **(C)** number of publications and H-index of countries/regions.

Over the past two decades, research on the relationship between migraine and CGRP has involved 1,315 organizations across 64 countries/regions. Table 1 presents the top 10 countries/regions and organizations contributing to this field. The United States leads with 2040 articles, followed by Italy (850) and China (557). These countries collectively account for over half of the total publications and represent core contributors to this field of study. Interestingly, 13 countries have contributed only one article each, while 41 countries have made more substantial contributions with at least five articles, highlighting variations across different regions. Analysis of total citation counts reveals that the USA (23,634 citations) has garnered the highest number of citations, followed by Denmark (6,491), Italy (5,419), UK (4,262), and Germany (3,308). Collaboration analysis reveals the USA’s prominence in multi-country collaborations (159 articles), followed by Italy (52) and China (26), highlighting potential for increased international cooperation, especially in China. Figure 3A illustrates active collaboration between different countries in Europe and North America, with developed countries primarily driving these collaborations. For example, the United States is at the centre of the cooperation, closely collaborating with Denmark, the United Kingdom, and Germany (Figure 3B). In contrast, China, a prominent country in Asia, demonstrates close collaboration with the USA, while other Asian countries generally cooperate less. The H-index, effective in predicting future research achievements and resource allocation, further underscores the leading contributions of specific countries in this field (36). Figure 3C displays the H-index related to neurology, with the USA leading at 536, followed by the United Kingdom (391), Germany (339), Canada (307), and Italy (270), highlighting their significant achievements and contributions to research on CGRP in migraine.

**Table 1:** The top 10 countries/regions and organizations contributed to publications in CGRP-related research in migraine.

Rank	Country	Articles	MCP	TC	H-index	Affiliation	Location	Articles	SCImago Institutions rankings
1	United States	2040	159	23,634	536	University of Copenhagen	Denmark	328	86
2	Italy	850	52	5,419	270	Erasmus Medisch Centrum	Netherlands	88	74
3	China	577	26	1898	174	Harvard University	United States	87	1
4	Denmark	404	86	6,491	184	Mayo Clinic	United States	87	10
5	Germany	347	40	3,308	339	King’s College London	United Kingdom	79	49
6	Spain	249	38	3,042	205	University of London	United Kingdom	78	254
7	United Kingdom	245	39	1898	391	University of Iowa	United States	77	192
8	Netherlands	187	8	1767	268	University of California	United States	75	16
9	Japan	124	31	1,160	216	Humboldt-Universitat zu Berlin	Germany	72	1,549
10	Hungary	105	12	748	105	Charite - Universitatsmedizin Berlin	Germany	71	95

MCP, multiple country publications; TC, total number of local citations.

**Figure 4:**
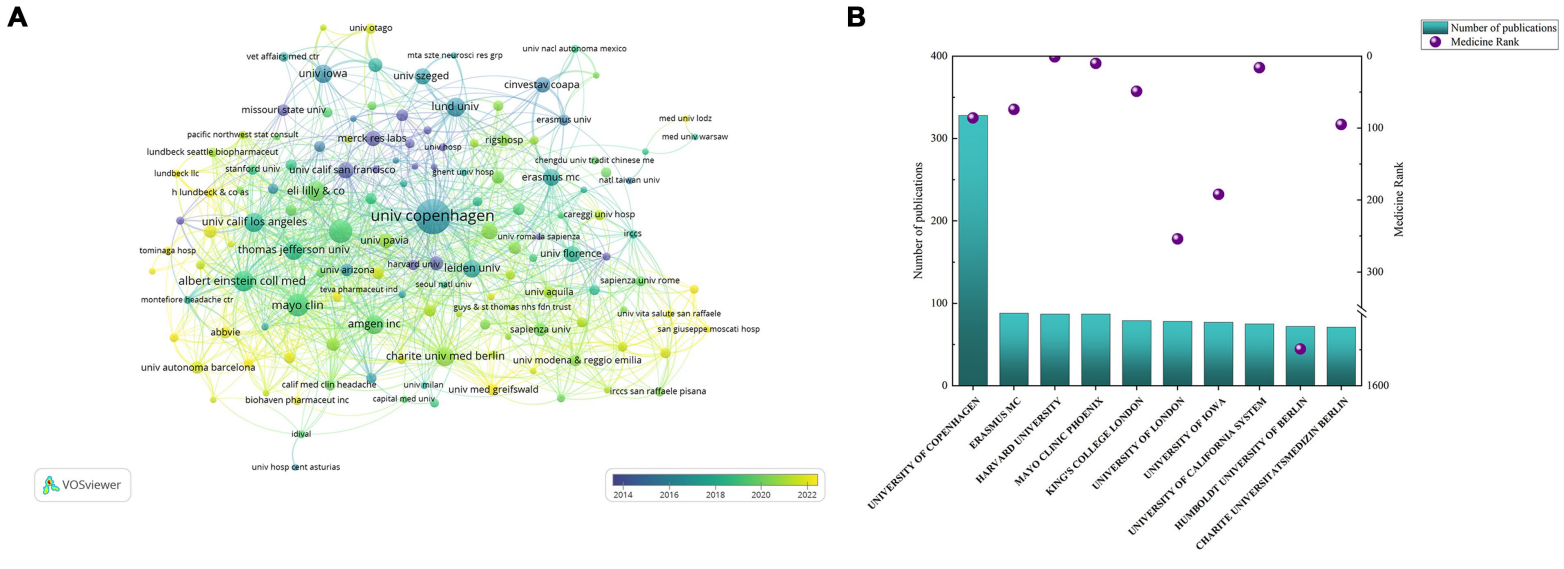
Cooperation maps of institutions in CGRP-related research in migraine: **(A)** co-occurrence network of institutions; **(B)** number of publications and SCImago rank of institutions.

Figure 4A illustrates the institutional cooperation network in the field of CGRP in migraine. The University of Copenhagen in Denmark has contributed significantly to this area with 328 published articles and maintains close collaborative relationships with institutions such as Mayo Clinic (87 publications) in the USA and Lund University (47 publications) in Sweden. Furthermore, notable collaborations are observed with Erasmus Medisch Centrum in the Netherlands, Harvard University in the USA, and King’s College London in the UK, which have published 88, 87, and 79 papers, respectively. The SCImago Institutions Rankings offer a comprehensive assessment of scientific impact, considering publication output, high-quality publications, and the world average impact (37). Institutions like Harvard University (ranked 1st), Mayo Clinic (ranked 10th), Erasmus Medisch Centrum (ranked 74th), and the University of Copenhagen (ranked 86th) have made essential contributions within the field of Medicine subject areas (Figure 4B). It’s not difficult to see that the University of Copenhagen has demonstrated a longstanding and in-depth attention to this research area compared to other institutions. Supplementary Figure S1 shows the top 15 institutions with the strongest citation bursts.

### Analysis of journals and co-cited journals

**Figure 5:**
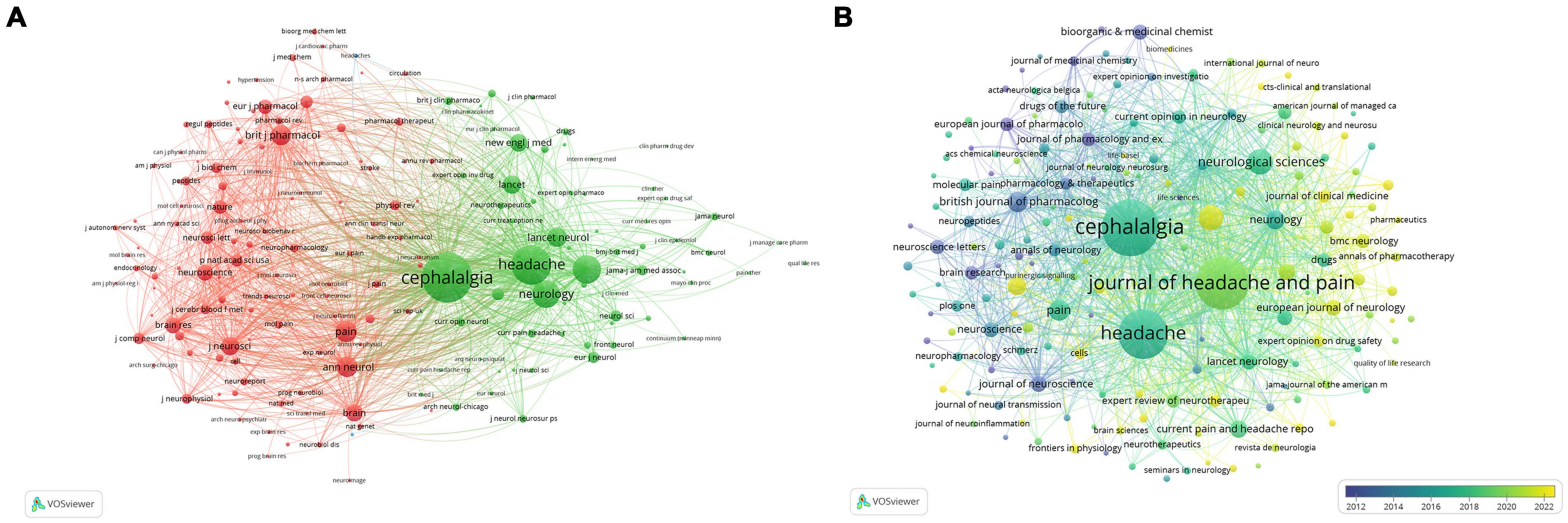
Cooperation maps of journals in CGRP-related research in migraine: **(A)** the visualization network of co-journals in CGRP for migraine; **(B)** the overlay network of journals according to the average years of publication.

Over the past 20 years, research on the relationship between CGRP and migraine has been published in 362 journals, with the top 10 most influential journals listed in Table 2. Upon scrutinizing the distribution of these papers, the top five journals collectively contributed 651 articles. *Cephalalgia* leads with 217 publications, followed by the *Journal of Headache and Pain* with 187 articles, and *Headache* ranking third with 162 articles. *Neurology* stands out with the highest Impact Factor (IF) among the top 10 journals at 10.1, with a contribution of 24 articles to this research domain. Among the top 10 academic journals, four are based in the United States, two in the United Kingdom, and two in Switzerland, underscoring the robust research foundations in these countries. Six journals (*Journal of Headache and Pain*, *Headache*, *Pain*, *British Journal of Pharmacology*, *Neurology*, and *International Journal of Molecular Sciences*) exhibit an Impact Factor > 5 and are considered of Q1 quality, indicating far-reaching development prospects for this field. Figure 5 illustrates the co-citation relationships between journals (Figure 5A) and the co-occurrence of journals (Figure 5B). The prominence of a journal within a specific research area is reflected by its number of co-citations. *Cephalalgia*, *Journal of Headache and Pain*, and *Headache* are consistently among the top three co-cited journals, suggesting closely aligned research topics across these publications. This shows the representativeness and persuasiveness of these journals in supporting research on CGRP-related in migraine. Furthermore, the SCImago Journal Rankings (SJR) provides insights into the scientific influence of journals, evaluating their prestige and impact based on weighted citations (38, 39). The H-index, a measure of a journal’s productivity and impact, quantifies the number of articles (h) with at least h citations (38). By integrating these indicators, journals like *Neurology*, *Pain*, *British Journal of Pharmacology*, *Cephalalgia*, and *Journal of Headache and Pain* emerge as journals of higher quality and significant impact in this research field compared to others.

**Table 2:** The top 10 productive journals related to CGRP in migraine.

Rank	Journals	Country	ND	TC	H-index	IF and JCR division (2022)	SCImago Journal Rank indicator
1	Cephalalgia	Norway	217	13,904	138	4.9, Q1	1.561
2	Journal of headache and pain	United Kingdom	187	4,497	79	7.4, Q1	1.593
3	Headache	United States	162	8,725	133	5.0, Q1	1.283
4	Neurological Sciences	Italy	44	745	83	3.3, Q2	0.765
5	Frontiers in Neurology	Switzerland	41	308	91	3.4, Q2	0.978
6	Pain	United States	30	2,614	282	7.4, Q1	2.445
7	British Journal of Pharmacology	United Kingdom	28	2,282	234	7.3, Q1	2.019
8	Neurology	United States	24	4,285	396	10.1, Q1	2.537
9	International Journal of Molecular Sciences	Switzerland	23	166	230	5.6, Q1	1.154
10	Current Pain and Headache Reports	United States	20	418	76	3.7, Q1	0.796

ND, number of documents; TC, total number of local citations.

### Analysis of cited references

Global citation is used to show the global academic impact of the articles in Web of Science, whereas local citations include only the reference list of articles included in the present bibliometric analyzed collection. Global citations reflect broader interdisciplinary influence and local citations provide insights into regional impact within the analyzed bibliometric collection (40). In this study, we focus on global citations sourced from the Web of Science database to evaluate the impact of CGRP-related research in migraine. Table 3 outlines the top 10 articles with the highest citation counts in this domain. The comprehensive dataset of 1,821 retrieved papers collectively amassed 56,999 citations. One hundred twenty-four articles (6.81%) garnered at least 100 citations, conversely, 162 articles (8.90%) did not receive any citations. The article by Jes Olesen published in the *New Engl Med* in 2004 (41) stands out as the most cited, with 927 global citations, followed closely by paper from Peter Goadsby, 2017, *Physiol Rev* (13) with 902 global citations and Fiona Russell, 2014, *Physiol Rev* (10) with 667 global citations. Jes Olesen, 2004, New Engl Med (41) is the most cited reference and document with the most local citations. Nine out of the top 10 referenced articles are included in our dataset, reflecting the relevance and coverage of our analysis. Supplementary Table S1 summarizes the top 10 most cited references spanning the period from 2004 to 2023.

**Table 3:** The top 10 publications on CGRP in migraine with the most citations.

Rank	Paper	DOI	Global citations	TC per year	Normalized TC	Local citations	LC/GC ratio (%)
1	Olesen J, 2004, New Engl J Med	10.1056/NEJMoa030505	927	46.35	6.99	510	55.02
2	Goadsby PJ, 2017, Physiol Rev	10.1152/physrev.00034.2015	902	128.86	15.00	218	24.17
3	Russell FA, 2014, Physiol Rev	10.1152/physrev.00034.2013	667	66.70	11.77	175	26.24
4	Goadsby PJ, 2017, New Engl J Med	10.1056/NEJMoa1705848	507	72.43	8.43	275	54.24
5	Edvinsson L, 2018, Nat Rev Neurol	10.1038/s41582-018-0003-1	489	81.50	9.77	244	49.90
6	Tepper S, 2017, Lancet Neurol	10.1016/S1474-4422(17)30083-2	447	63.86	7.44	277	61.97
7	Ho TW, 2008, Lancet	10.1016/S0140-6736(08)61626-8	447	27.94	6.08	261	58.39
8	Silberstein SD, 2017, New Engl J Med	10.1056/NEJMoa1709038	427	61.00	7.10	240	56.21
9	Ho TW, 2010, Nat Rev Neurol	10.1038/nrneurol.2010.127	373	26.64	6.15	202	54.16
10	Durham PL, 2004, Headache	10.1111/j.1526-4610.2004.04007.x	372	18.60	2.81	32	8.60

DOI, digital object identifier; TC, total citations; LC/GC, local citation/global citation.

**Figure 6:**
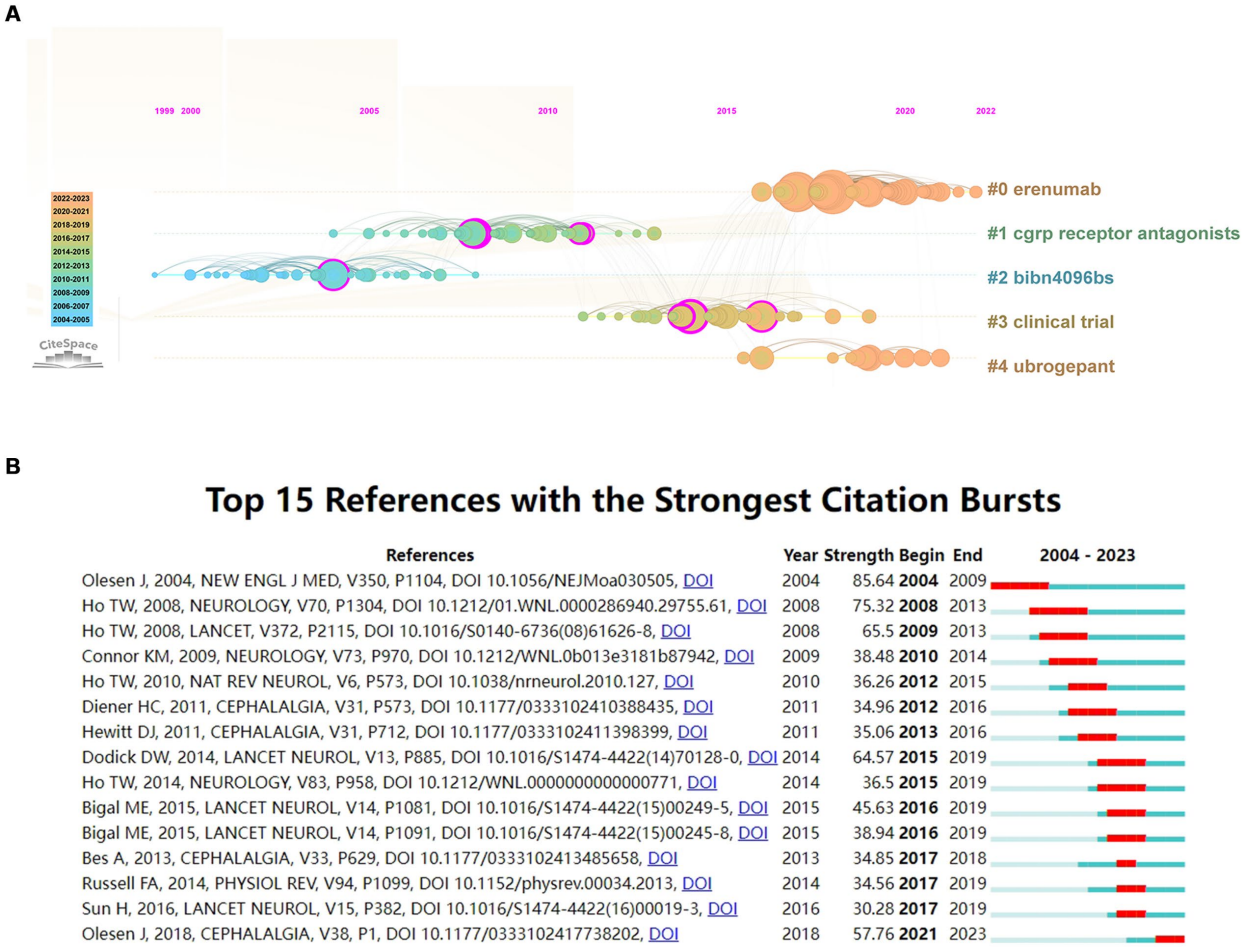
**(A)** Timeline view of co-citation reference. The timeline from left to right represents the time evolution from 2004 to 2023; **(B)** Top 15 references with strong citation bursts (A red bar indicates high citations in that year).

Noun phrases extracted from co-cited references were utilized to identify clusters representing research patterns and cutting-edge themes within the networks. Figure 6A depicts the evolution of four distinct clusters over a 20-year span. Cluster #1 focuses on CGRP receptor antagonists, while Cluster #2, centered around BIBN4096BS, initially garnered significant attention. However, over time, interest in these areas has waned due to the lack of commercialization and the cessation of development owing to hepatotoxicity. Cluster #3, emphasizing clinical trials, emerged as a predominant hotspot during the intermediate phase from 2004 to 2023, indicating substantial accumulation of clinical research experience in CGRP-related migraine studies. In contrast, Cluster #0 centered around erenumab and Cluster #4 focusing on ubrogepant have experienced recent peaks in interest and continue to be subjects of ongoing study. The *Q* value of 0.6107 (>0.3) and *S* value of 0.9003 (>0.5) derived from this study indicate the credibility and reliability of our findings.

This study utilized CiteSpace to identify 15 with exhibiting strong citation bursts (Figure 6B), characterized by sudden increases in citations and widespread attention over specific time periods (42). The observed citation bursts spanned from as early as 2004 to as recently as 2021, highlighting enduring impact and continued relevance of certain references in this field. Among the identified references, Jes Olesen, 2004, *New Engl Med* (41) exhibited the strongest citation burst (strength = 85.64) from 2004 to 2009. Similarly, Ho Tony, 2008, *Neurology* (43) (strength = 75.32) demonstrated a strong citation burst (strength = 75.32) from 2008 to 2013. The burst strengths of these influential references ranged from 30.28 to 85.64, with endurance strength lasting between 1 and 5 years. Table 4 provides a summary of the primary research contents of these 15 references, organized in the sequence presented in Figure 6B.

**Table 4:** The Main Research Contents of the 15 References with Strong Citations Bursts.

Rank	**Strength**	**Main research content**
1	85.64	A randomised controlled clinical trial of CGRP receptor antagonist BIBN4096BS for the acute treatment of migraine confirmed its efficacy and safety (41).
2	75.32	CGRP receptor antagonist, MK-0974, was confirmed its effective and generally well tolerated for the acute treatment of migraine by a randomised controlled trial (43).
3	65.50	A new oral antagonist of CGRP receptor MK-0974 (telcagepant) is effective as an acute treatment for migraine with fewer associated adverse effects by a randomised, placebo-controlled, parallel-treatment trial (44).
4	38.48	This large Phase 3 clinical trial confirmed that the first oral CGRP receptor antagonist (telcagepant) was effective at relieving pain at 2 hours and pain freedom for up to 24 hours, and generally well tolerated (45).
5	36.26	CGRP and its receptors were highlighted, which provide new insights into migraine pathophysiology (46).
6	34.96	A phase II study demonstrated the oral CGRP receptor antagonist (BI44370TA) was shown in a dose-dependent manner in the treatment of acute migraine attacks (47).
7	35.06	CGRP receptor antagonist MK-3207 was effective and generally well tolerated in the acute treatment of migraine by a randomised controlled trial (48).
8	64.57	This phase 2 study demonstrates that LY2951742, a fully humanised monoclonal antibody to CGRP, may be beneficial in migraine prevention and provides support for the role of calcitonin gene-related peptides in migraine pathogenesis (49).
9	36.50	This study provides Class II evidence that in patients with migraine, telcagepant taken daily reduces headache days compared to placebo, but the observed aminotransferase elevations do not support the use of telcagepant for daily administration (50).
10	45.63	This trial assesses the safety, tolerability, and efficacy of TEV-48125, a monoclonal anti-CGRP antibody, in the preventive treatment of high-frequency episodic migraine, which supports the phase 3 clinical trials (51).
11	38.94	This phase 2b study established the safety, tolerability, and efficacy of a monoclonal anti-CGRP antibody (TEV-48125) in chronic migraine and support phase 3 trials (52).
12	34.85	ICHD-3 (beta version), A set of detailed and standardized diagnostic criteria for use by clinicians in their practice, to allow for additional testing prior to the finalisation of ICHD-3 (53).
13	34.56	Physiological and pathological interpretation and discussion of calcitonin gene-related peptides (10).
14	30.28	This phase 2 trial suggests that a fully humanised monoclonal antibody to CGRP receptor (AMG334) might be a potential therapy for prevention in patients with episodic migraine and support further investigation in larger phase 3 trials (54).
15	57.76	ICHD-3, which better distinguishes headache from other brain disorders, adds new headache-related features and lays the foundation for future revisions of the ICDH (1).

### Analysis of authors

**Figure 7:**
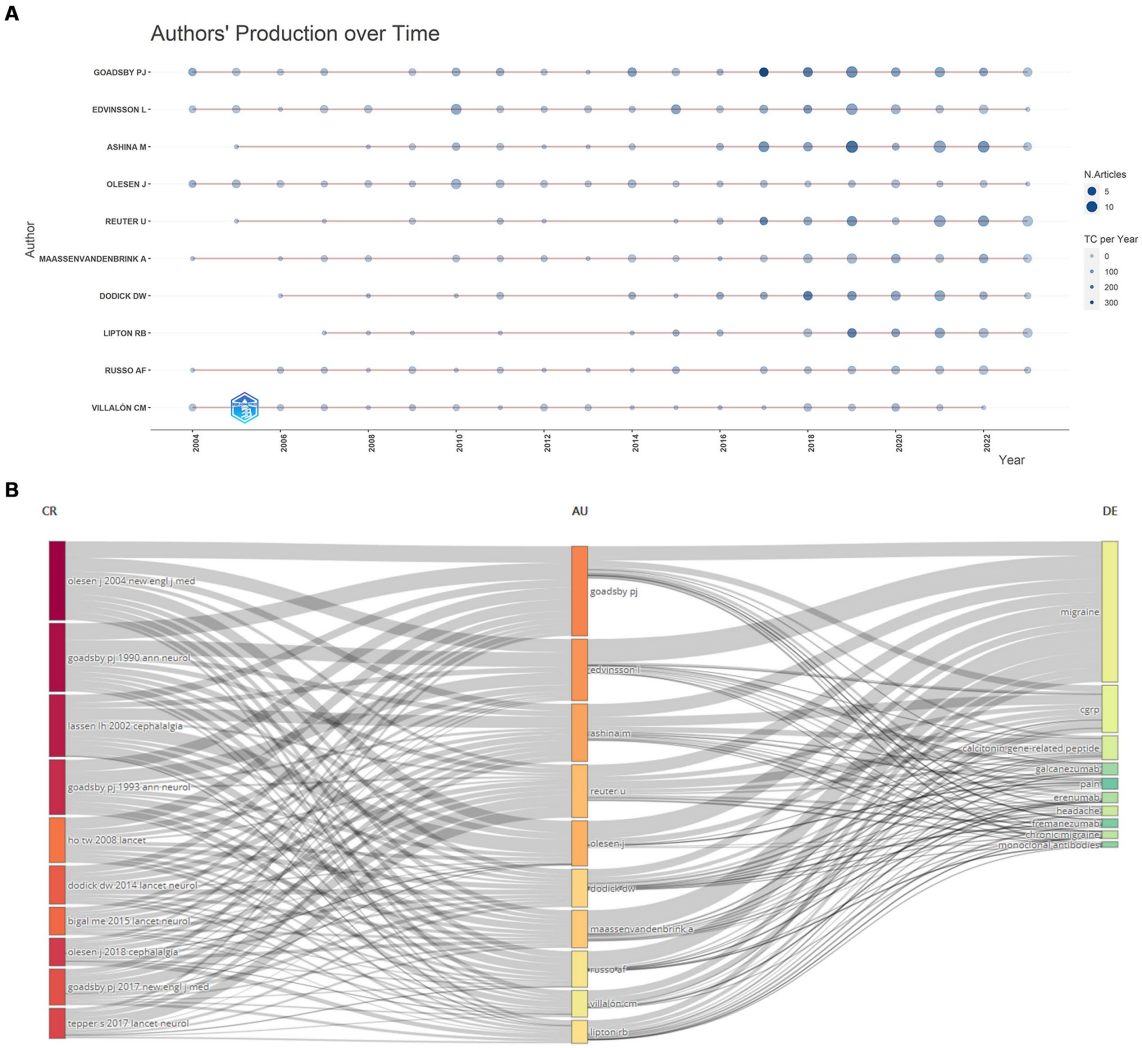
**(A)** Author’s scientific production over time; **(B)** three-field plot between top authors, research fields, and reference citations (in LCS).

A total of 4,752 authors contributed to the publication of 1,821 papers in CGRP-related research in migraine. Table 5 presents the top 10 most cited authors in this domain, with seven authors contributing at least 50 papers each, while a large number of authors (3,585) contributed a single article each. Leading the list is Peter Goadsby with 9,733 global citations, 4,608 local citations, and 89 publications, followed by Lars Edvinsson with 5,227 global citations, 2,849 local citations, and 84 publications, ranking second. Messoud Ashina holds the third position. Supplementary Figure S2 illustrates the central role of Peter Goadsby, Lars Edvinsson, and Messoud Ashina in collaborative networks within the field of CGRP-related research in migraine. Scientific impact is further assessed using metrics such as the H-index, which measures the number of citations received for published papers (55). The G-index, a derivative of the H-index, can average citations from highly cited articles over a larger number of articles to some extent (56). Figure 7A visually represents production over time and citation impact, with circle size indicating publication volume and color intensity reflecting the number of citations. Figure 7B depicts the relationship between top authors, research fields, and reference citations (in LCS), highlighting the overall expertise and contributions within specific areas of study. These findings underscore the significant contributions and collaborative efforts of leading authors like Peter Goadsby, Lars Edvinsson, and Messoud Ashina in advancing CGRP-related research in migraine.

**Table 5:** The top 10 most influential authors based on the global citations.

Rank	Author	Global citations	Local citations	NP	H-index	G-index	Articles fractionalized
1	Goadsby PJ	9,733	4,608	89	47	89	26.49
2	Edvinsson L	5,227	2,849	84	37	71	29.23
3	Ashina M	4,573	2,459	80	35	67	14.38
4	Olesen J	3,893	2,064	64	31	62	15.88
5	Dodick DW	4,749	2,818	50	28	50	7.35
6	Reuter U	3,375	2,013	61	27	58	10.09
7	Russo AF	2,208	984	46	26	46	13.86
8	Lipton RB	2,507	1,349	47	25	47	6.95
9	Maassenvandebrink A	1,662	814	60	23	39	12.45
10	Jansen-Olesen I	810	385	28	19	28	5.48

NP, number of publications.

### Analysis of keywords

**Figure 8:**
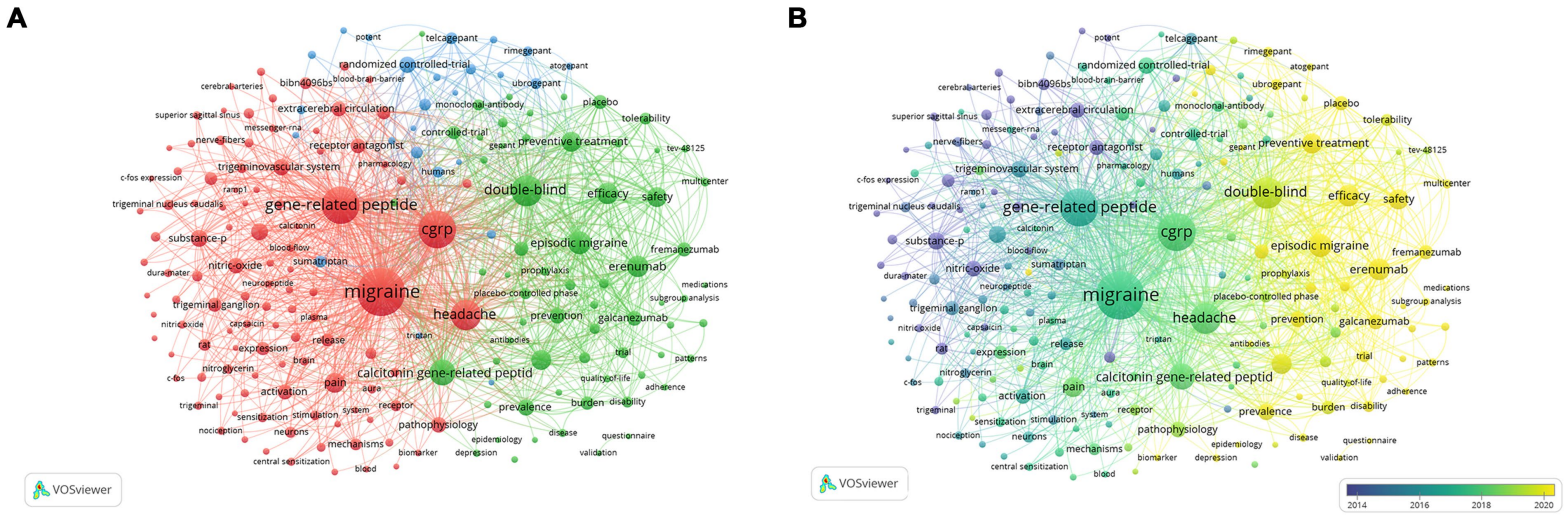
Co-occurrence network analysis of keywords regarding CGRP in migraine study: **(A)** the keywords cluster analysis; **(B)** visualization of the keyword co-occurrence network according to the average years of publication.

This study extracted 2,461 keywords from 1,821 articles published in the last 20 years. The top 10 keywords plus and author keywords are depicted in Supplementary Table S2. Using CiteSpace and VOSviewer to analyze keywords co-occurrence and clustering can help understand the research progress and highlight, and using trend topics to describe the terms being in trend can help understand the research hotspots and frontiers in this field. Keyword co-occurrence analysis using VOSviewer revealed three distinct clusters representing different research directions (Figure 8A). Cluster #1 (red) primarily focuses on studying migraine pathogenesis, CGRP and its receptor mechanisms. Cluster #2 (green) emphasizes indicators and criteria for assessing clinical trials related to migraine. Cluster #3 (blue) centers on clinical trials of CGRP antagonists for migraine treatment. Specific keywords within these clusters include ‘migraine,’ ‘CGRP,’ ‘headache,’ and ‘trigeminovascular system’ in the red cluster; ‘double-blind,’ ‘calcitonin gene-related peptide,’ ‘efficacy,’ ‘safety,’ and ‘preventive treatment’ in the green cluster; and ‘randomized controlled-trial,’ ‘monoclonal-antibody,’ and specific drug names like ‘telcagepant,’ ‘ubrogepant,’ ‘atogepant,’ and ‘rimegepant’ in the blue cluster. The keywords were coded into different color types per the latest average appearing year (AAY) of publication (Figure 8B). Research from 2004 to 2016 predominantly focused on CGRP pathogenesis and therapeutic potential in migraine. Since 2017, there has been active exploration and development of CGRP antagonists, accompanied by basic research and clinical trials. In recent years (2021–2023), frequent keywords with the latest AAY indicate a surge in large-scale clinical applications and novel developments of CGRP receptor antagonists and monoclonal antibodies, signifying current research hotspots and key directions for future investigations.

The top 15 keywords with the strongest citation bursts were identified using CiteSpace (Supplementary Figure S3). Noteworthy keywords such as nitric oxide (26.11, 2004–2016), substance P (25.89, 2004–2015), receptor antagonist (20.95, 2004–2015), and extracerebral circulation (17.12, 2004–2009) represent the foundation and the starting point of migraine treatment research over the past two decades. In contrast, keywords like CGRP receptor antagonist (10.83, 2011–2016), randomized controlled trial (12.57, 2007–2016), placebo (11.48, 2020–2023), and trial (11.84, 2021–2023) reflect the increasing emphasis on evaluating the clinical efficacy of CGRP receptor antagonists in migraine management. These findings highlight a shift in research focus towards exploring and validating novel therapies in this field. Supplementary Figure S4 shows the temporal trends of keywords plus and author keywords related to CGRP in migraine. The observed trends are consistent with the results obtained from keyword co-occurrence analysis using VOSviewer and citation burst analysis conducted with CiteSpace.

## Discussion

This study represents the first comprehensive bibliometric analysis aimed at uncovering the current status and future trends in CGRP-related research within the context of migraine. A systematic search of the Web of Science Core Collection (WoSCC) database retrieved 1,821 papers and reviews published between 2004 and 2023, as of December 31, 2023. The distribution across countries and institutions, journal quality, and author contributions of these 1,821 articles were meticulously assessed using analysis and visualization tools, including CiteSpace, VOSviewer, and the R package “bibliometrix.” Additionally, we employed citation burst analysis, cluster analysis, and keyword analysis to identify the current hotspots of focus and emerging trends in CGRP-related research in migraine.

### General information

A total of 1,821 papers, accruing 56,999 citations, were published in 362 journals by 1,315 organizations spanning 64 countries/regions. From 2004 to 2016, the research output fluctuated with an average annual publication rate of 42.5 papers. From 2017 to 2022, the field saw rapid growth, with an average of 183.5 papers published annually, indicating a substantial increase in researchers’ attention towards CGRP in migraine. Despite a slight decrease in 2023, there has been a steady increase in CGRP-related migraine studies. This ascending trend highlights the topic’s significance and its potential future impact on migraine treatment. Our analysis identified notable shifts in CGRP-related migraine research focus over time. Initially, research emphasized the potential role of CGRP in inducing inflammation and vasodilation in migraine pathogenesis. Subsequently, investigations increasingly delved into the intricate signaling pathways underlying CGRP-mediated inflammation in migraine. Future research is expected to explore and develop novel anti-CGRP drugs for migraine treatment, prioritizing large-scale clinical applications of CGRP receptor antagonists and monoclonal antibodies. This shift underscores a move towards translational research and therapeutic innovation. Additionally, the number of annual publications in this field is projected to increase further.

The number of publications on CGRP-related migraine research in the top five countries has consistently increased since 2004. Significant contributions come from the United States, Italy, China, Germany, and Denmark. After a period of initial absence from 2004 to 2006, China’s output notably grew from 2009, surpassing Denmark and Germany by 2019, indicating rapid development. The United States leads in publications, citations, and H-index, indicating its central role and high-quality research. Figure 3B illustrates that collaboration is primarily seen among developed nations in Europe and North America, with strong cooperation between China and the United States. In contrast, there are fewer partnerships with other countries. This disparity can be attributed to varying levels of societal awareness about migraine’s impact and differential investment in research funding across countries. Developing nations often face challenges such as brain drain and technological limitations due to resource constraints, which contribute to disparities in research productivity compared to developed countries (57, 58). To address these challenges and foster advancements in CGRP-related migraine research, it is imperative for scholars across countries to break academic boundaries and engage in active communication and collaboration.

The University of Copenhagen stands out as the institution with the highest number of publications and boasts a strong ranking in the SCImago Institutions Rankings, reflecting its significant contributions to CGRP-related research in migraine. Other prominent institutions such as Mayo Clinic, Lund University, Erasmus Medisch Centrum, and Harvard University also demonstrate active engagement in this field and maintain collaborative relationships. The collective efforts of these institutions indicate their key role in advancing our understanding of CGRP in migraine. An analysis of the top 10 institutions by publication volume reveals that 40% are based in the United States, highlighting the country’s substantial influence in this research domain. Merck & Company exhibits the highest outbreak intensity, indicating a significant output and contribution to CGRP in migraine during the period from 2006 to 2016. Lund University and Missouri State University demonstrate the longest citation burst duration, suggesting their profound impact and sustained influence in the field of CGRP in migraine.

The journal with the highest impact factor is *Neurology* (*IF* = 10.1, Q1), followed by *Pain* (*IF* = 7.4, Q1), and *Journal of Headache and Pain* (*IF* = 7.4, Q1), all of which published notable research, including high-quality clinical trials related to migraine (59–61). The majority of CGRP-related research in migraine was concentrated in journals like *Cephalalgia* (*IF* = 4.9, Q1), *Journal of Headache and Pain* (*IF* = 7.4, Q1), and *Headache* (*IF* = 5.0, Q1), indicating they made significant contributions and can be considered currently the most popular journals in this field. *Cephalalgia*, *Journal of Headache and Pain*, and *Headache* are specialized journals focusing on headache disorders, emphasizing clinical and therapeutic advancements. They are primary sources for studies on the clinical and pathological aspects of migraines, as evidenced by their high publication and co-citation rates. In contrast, journals like *Neurology*, *British Journal of Pharmacology*, *Pain*, and *International Journal of Molecular Sciences* cover broader topics in neurology, pharmacology, and molecular mechanisms, including migraine-related neurological mechanisms, pharmacodynamics of anti-migraine drugs, and CGRP’s molecular role in migraines. By analyzing between headache-specific journals and those with broader neurological and pharmacological scopes, we can better appreciate the multidisciplinary nature of migraine and CGRP research. In summary the current research on CGRP in migraine is published in high-quality journals, indicating that it is currently in the stage of deepening basic research with wide clinical application. The sustained interest and attention garnered by CGRP-related research in migraine over recent years indicate that the field is poised for continued and sustainable development, emphasizing the importance and potential impact of CGRP in migraine treatment.

The assessment of a researcher’s expertise and collaborative potential in a specific field can be determined by the number of high-quality publications and citations. Our analysis identified the top 10 authors with the most global citations in CGRP-related migraine research. Peter Goadsby from King’s College London leads, followed by Lars Edvinsson, also from Lund University, and Messoud Ashina from the University of Copenhagen. The visual network of authors indicates close collaboration among these researchers, highlighting their collaborative potential in this research area. Notably, five authors began publishing in 2004, with Peter Goadsby and Jes Olesen co-authoring the highest-cited article (927 citations) in the *New England Journal of Medicine* (*IF* = 158.5), which conducted a pivotal randomized controlled clinical trial of the CGRP receptor antagonist BIBN4096BS for acute migraine treatment. This trial demonstrated the efficacy and safety of BIBN4096BS, foundational for subsequent research and clinical use (41). Another influential review article (902 citations) by Antoinette MaassenVanDenBrink and Carlos Villalon in the *European Journal of Pharmacology* (*IF* = 5.0) discussed CGRP’s role in migraine pathophysiology and treatment modalities (22). An experimental study (667 citations) by Jes Olesen and Lars Edvinsson in the *British Journal of Pharmacology* (*IF* = 7.3) confirmed BIBN4096BS’s inhibitory action on large dural blood vessels in rats, implicating dural blood vessels in migraine pathogenesis (62). From 2010 to 2023, the publication output and annual citations of Peter Goadsby, Jes Olesen, and Lars Edvinsson remained relatively stable. In addition, Messoud Ashina’s publications have increased steadily since 2016, particularly through participation in clinical trials involving various anti-CGRP drugs, providing critical evidence in this field (63–65). Thus, Messoud Ashina emerges as a prolific author and promising collaborator in CGRP-related migraine research, significantly advancing the understanding and treatment of migraine through CGRP-related mechanisms.

### Knowledge base

Reference citations and co-citations play a crucial role in uncovering the knowledge base of relevant research areas, providing insights into research progress, and identifying key issues to address. Cluster analysis offers a valuable approach to identifying stage-specific hotspots, tracking progress, and outlining future directions within a research domain by analyzing different clusters chronologically. In this study, we have assessed the knowledge base of migraine research through the top 10 cited references and typical cluster analysis. Among these references, the most influential paper identified By GCS (ranked 1 for LCS) has been detailed previously. The article on BIBN4096BS (Olcegepant), the first potent and selective nonpeptide calcitonin gene-related peptide 1 (CGRP1) receptor antagonist, stands out as a significant contribution to the field of CGRP receptor antagonists in migraine (41). This pioneering work has demonstrated the efficacy and tolerability of BIBN4096BS as an anti-migraine drug, making substantial impacts evident through its sustained high citations within the field.

The second and fourth most influential articles in GCS (ranked 9 and 4 for LCS) were authored by Peter Goadsby and published in *PHYSIOL REV* (*IF* = 33.6, Q1) and *NEW ENGL J MED* (*IF* = 158.5, Q1) in 2017, respectively. The former paper titled “Pathophysiology of Migraine: A Disorder of Sensory Processing” elucidates the clinical manifestations of migraine and illustrates the relevant anatomy, physiology, and pharmacology of these symptoms (13). This comprehensive review highlights the broad impact on brain function when migraine undergoes systemic changes, thus supporting theories related to migraine pathogenesis. The latter paper titled “A Controlled Trial of Erenumab for Episodic Migraine” presents findings suggesting that erenumab, a fully human monoclonal antibody, may be effective in preventing episodic migraine (66). This study provides valuable clinical evidence for the potential use of CGRP monoclonal antibodies in migraine treatment.

The article titled “Calcitonin gene-related peptide: physiology and pathophysiology” authored by Fiona Russell has been identified as the third most influential publication in the literature based on GCS (10). Although it does not appear in the top 10 list of the LCS, this paper remains noteworthy and deserving of reference. In this review, Fiona Russell comprehensively describes CGRP, covering its structure, synthesis, release, and metabolism, and discusses its physiological and pathological actions in various contexts. The review also explores the potential indications for CGRP receptor antagonists currently under development for migraine treatment. This study summarizes the diverse roles CGRP may play in different conditions, helping researchers provide subsequent insights into the long-term effects of depletion of CGRP levels in humans, especially in migraine.

The fifth-ranked article in the GCS, ranked sixth for LCS, was published in 2018 by Lars Edvinsson in *Nat Rev Neurol* (*IF* = 38.1, Q1). During this period, the treatment of migraine entered a new era with the development of drugs targeting CGRP or its receptors. Several drugs specifically designed to target the trigeminal sensory neuropeptide, known as CGRP receptor antagonists, were expected to be approved for use in migraine in 2018 and 2019. This article summarizes the key clinical evidence for the role of CGRP in migraine and provides an overview that contributed to the successful development of CGRP receptor antagonists (25).

The sixth, seventh, and eighth most influential papers, as ranked by the GCS and third, fifth, and seventh in the LCS, were all randomized, double-blind, placebo-controlled clinical studies of CGRP receptor antagonists and monoclonal antibodies for migraine in adults, including erenumab, telcagepant, and fremanezumab (44, 63, 67). These three pivotal clinical trials have accelerated the translation of CGRP-related research in migraine from theory to clinical application, establishing a robust foundation for subsequent drug marketing and widespread use, and have become standards for future investigations. Of particular note, telcagepant (MK-0974) is an oral medication, while the other two are administered via subcutaneous injections. A clinical study evaluating telcagepant’s effectiveness for migraine prevention in 2014 was terminated prematurely by the Safety Monitoring Board due to hepatotoxicity concerns among patients (50). The adverse reaction was believed to be closely related to the treatment’s frequency and duration. As a result, telcagepant was not recommended for daily administration in chronic migraine treatment.

The ninth and tenth most impactful articles within the GCS did not feature in the top 10 list of the LCS. The former is a comprehensive review providing a systematic overview of the role of CGRP and examining neuronal mechanisms in migraine development. Traditionally, the field of migraine was dominated by the vascular theory and the central neuronal theory. This review integrated available evidence to demonstrate that triggers for migraine pathogenesis are more focused on disturbances in the overall balance of circuits involved in sensory modulation rather than solely on vasodilation, providing new insights into migraine pathophysiology (46). As early as 1998, botulinum toxin was investigated for treating pericranial myalgia triggered by migraine, although evidence for its efficacy during acute migraine episodes has been inconclusive (68). The latter study was a fundamental experimental investigation confirming that botulinum toxin type A’s efficacy in migraine treatment may stem from its ability to reduce CGRP levels from trigeminal neurons (69).

A cluster analysis of the cited references from the 1,821 articles was conducted to identify homogeneous clusters of highly co-cited publications related to CGRP in migraine. Cluster #2, centered around BIBN4096BS (olcegepant), marks the first nonpeptide CGRP receptor antagonist effective in clinical trials, representing a significant pharmacological advancement in migraine treatment post the advent of triptans in the early 1990s (70). During this period, numerous basic experiments and clinical trials have shown that BIBN4096BS may act on trigeminal ganglion neurons, skull meningeal, and the middle cerebral artery, thereby exerting corresponding effects (41, 71, 72). Cluster #1 focuses on CGRP receptor antagonists, highlighting the rationale and validation of these agents in migraine treatment. mRNA studies reveal widespread CGRP distribution in trigeminovascular and other pain processing structures, suggesting its role in migraine pathogenesis (73). While acute migraine treatment in clinical practice has been dominated by triptans, which carry cardiovascular side effects, gepants, a class of CGRP receptor antagonists, represent a potential non-vasoconstrictive alternative. Researchers have continued to develop and validate various specific CGRP receptor antagonists and anti-CGRP monoclonal antibodies, including both oral and subcutaneous formulations, with notable examples such as telcagepant, fremanezumab, rimegepant, and MK-8825 (43, 74–76). Cluster #3, centered on clinical trials, indicates the extensive phase II and phase III trials conducted in the development of CGRP receptor antagonists and anti-CGRP monoclonal antibodies. Based on substantial preliminary pharmacological evidence, the translation of anti-CGRP drugs treatment for migraine from bench to bedside represents a methodical developmental process in scientific research. High-quality clinical trials have confirmed the importance of CGRP in migraine pathogenesis, laying a robust foundation for further research and fostering new avenues for novel anti-CGRP drugs. Current research on CGRP in migraine has particularly focused on drug development, emphasizing the evaluation of these drugs’ safety profiles alongside their demonstrated efficacy, as encapsulated in the articles covered in cluster #0 (Erenumab) and cluster #4 (Ubrogepant). The evolution of CGRP-related migraine research has progressively refined research clusters, advancing from elucidating CGRP mechanisms to assessing the efficacy and safety of CGRP receptor antagonists and monoclonal antibodies. This progression underscores the field’s methodical and stable development.

### Hotspots and frontiers

In this analysis, we describe the top 15 references exhibiting strong citation bursts identified by CiteSpace. These publications have garnered considerable attention from researchers at specific periods, reflecting key themes in the evolving landscape of this research area. We can find that the pursuit of standardized diagnostic criteria for migraine, exploration of CGRP and its receptor mechanisms in migraine pathogenesis, and evaluation of CGRP receptor antagonists and monoclonal antibodies for migraine treatment efficacy and safety emerge as predominant focal points in the field.

Furthermore, in addition to citation bursts, keywords serve as a reflection of article content, aiding in the swift identification of research hotspots within the CGRP in migraine. Focusing on keywords associated with disease types demonstrates an increased interest in understanding migraine pathogenesis, particularly in two types: episodic migraine and chronic migraine. Recent trends, reflected in keywords over the past 3 years, emphasize topics including migraine, CGRP, double-blind, chronic migraine, monoclonal antibodies, preventive treatment, efficacy, and erenumab. These trends suggest a growing recognition of the benefits of CGRP antagonists for migraine treatment, driving focused efforts toward the development and clinical application of CGRP receptor antagonists and monoclonal antibodies.

Keyword co-occurrence analysis and burst intensity analysis are instrumental in exploring research hotspots and frontiers within the specific field. Recent trends reflected in the average appearing year (AAY) keywords from Figure 8B highlight critical directions for future research in CGRP and migraine treatment. Keywords such as “episodic migraine,” “efficacy,” “erenumab,” “preventive treatment,” “safety,” “placebo,” “galcanezumab,” “ubrogepant,” and “rimegepant” signify areas of emphasis and potential growth in CGRP-related migraine research. The emergence of therapies targeting CGRP represents a transformative shift in migraine treatment. Two primary classes of drugs, anti-CGRP monoclonal antibodies (mAbs) and CGRP receptor antagonists (gepants), are now available as migraine-specific preventive treatments (11). The development of early CGRP receptor antagonists and anti-CGRP monoclonal antibodies faced significant setbacks. Telcagepant was discontinued in 2011 due to liver toxicity observed in long-term safety studies (44). Olcegepant’s development was halted primarily due to poor oral bioavailability (77). Similarly, the development of MK-3027 and MK-8825 was halted in the late 2000s because of liver toxicity concerns (48). Despite these challenges, subsequent anti-CGRP monoclonal antibodies like erenumab, fremanezumab, and galcanezumab have demonstrated better safety and efficacy, while gepants, including rimegepant and atogepant, have also been approved for migraine prevention. Rimegepant, in particular, serves a dual role. It provides effective relief for acute migraine attacks and is also utilized for preventive therapy, ensuring comprehensive management of migraine symptoms. These medications have demonstrated both efficacy and safety in clinical trials for episodic and chronic migraine, offering valuable real-world evidence that will inform future research directions in migraine treatment.

In the analysis of keyword burst intensity, keywords like “Nitric oxide,” “Substance P,” and “Receptor antagonist” exhibit strong burst intensity lasting more than 10 years. Over the years of migraine research, the latest scientific hypothesis has suggested the activation of the trigeminovascular system as a key factor in migraine attacks. However, the pathogenesis of migraine remains complex and multifaceted, with many aspects still awaiting exploration. CGRP is recognized as a pivotal mediator in migraine pathogenesis, playing a critical role in trigeminovascular system activation. Changes in CGRP levels can influence downstream signaling pathways following trigeminovascular system activation. Upon activation, trigeminal sensory nerve fibers release neuropeptides and vasoactive substances including CGRP, substance P (SP), and nitric oxide (NO) (78). This release characterizes neurogenic inflammation, accompanied by plasma protein extravasation (79). Experimental evidence has highlighted substance P as a key mediator of plasma protein leakage and vasodilation (80). NO, a potent vasodilator, can be activated by CGRP through endothelial NOS (eNOS), contributing to vasodilation through NO-mediated mechanisms (81, 82). A single subcutaneous injection of nitroglycerin (NTG) can activate brain regions associated with migraines, such as the trigeminal nucleus caudalis (TNC), leading to central trigeminocervical neuron sensitization (83, 84). Consequently, the NTG experimental model has become a preferred and widely utilized approach in CGRP-related migraine research. Many migraine medications may exert therapeutic effects by modulating NO- and CGRP-mediated pathways, reflecting the ongoing exploration and potential targets in migraine treatment.

In summary, CGRP plays a crucial role in the development of migraine and represents a promising therapeutic target for migraine treatment. Understanding the mechanisms involving CGRP and its receptors in migraine development can shed light on trigeminovascular system disorders. Furthermore, CGRP can be used as a biomarker to assist in the diagnosis of migraine. CGRP antagonists offer distinct advantages, including cardiovascular safety and sustained efficacy compared to commonly used triptans, making them valuable candidates for migraine treatment. Exploring and refining therapeutic approaches of anti-CGRP hold significant promise for improving migraine treatment outcomes (85). The transition from theoretical research to clinical practice underscores the importance of demonstrating the safety and efficacy of novel therapies. High-quality double-blind, randomized, controlled clinical trials serve as the gold standard for evaluating migraine treatment efficacy and safety. Moving forward, studies in this field should prioritize conducting more standard, high-quality clinical trials to assess the safety and efficacy of novel drugs and gain deeper insights into the pathophysiological mechanisms involving CGRP in migraine.

### Strengths and limitations

To the best of our knowledge, this study represents the first scientometrics-based bibliometric examination of the development and trends of CGRP in migraine. However, several potential limitations should be acknowledged in our study. Firstly, it’s important to note that bibliometric analysis can only reflect the current state of research in a particular field to a certain extent and cannot replace traditional systematic review. Secondly, the scope of our study was limited to English-language papers indexed in the WoSCC database. This approach may have excluded relevant studies published in other languages or indexed in different databases. Finally, a lack of keywords or abstracts in prior literature may increase the likelihood of being excluded due to poor discoverability. Future research could broaden the literature coverage by including additional databases such as PubMed and country-specific repositories to ensure a more comprehensive analysis of the field.

Despite its limitations, bibliometric analysis offers valuable insights into the essential role and extensive body of studies on CGRP in the context of migraine pathogenesis. Our study clearly displayed the countries/regions, institutions, journals, references, authors, keywords, and other aspects of literature related to CGRP in migraine over the past two decades. By collecting and reviewing datasets and publications from leading authors in the field, we gained a comprehensive understanding of the development process, current research status, and knowledge hotspots of CGRP in migraine. Through keyword analysis, we outlined future research directions and hotspots for CGRP in migraine, which can serve as a reference for researchers. We believe that this bibliometric research can provide valuable inspiration and ideas for further exploration in related fields.

## Conclusion

The bibliometric analysis of CGRP-related migraine research from 2004 to 2023 reveals a consistent increase in publications, with notable contributions from the United States, Italy, China, Denmark, and Germany, reflecting a global interest in this research area. Leading institutions include the University of Copenhagen, Erasmus Medisch Centrum, Harvard University, and Mayo Clinic. Key journals such as Cephalalgia and Neurology play pivotal roles in disseminating research findings. Prominent researchers like Peter Goadsby, Lars Edvinsson, and Messoud Ashina are highly influential. Major research themes encompass pathophysiological mechanisms, diagnostic criteria, preventive treatments, and anti-CGRP drugs. The study underscores the need for enhanced global collaboration among countries, institutions, and researchers in CGRP-related migraine research, aiming to optimize study designs, improve treatments, and benefit patients worldwide.

## Data availability statement

The raw data supporting the conclusions of this article will be made available by the authors, without undue reservation.

## Author contributions

LW: Writing – original draft, Methodology, Visualization, Conceptualization, Formal analysis, Writing – review & editing. QW: Formal analysis, Visualization, Investigation, Software, Writing – review & editing. HD: Visualization, Investigation, Software, Writing – review & editing. XL: Visualization, Investigation, Writing – review & editing. YZ: Funding acquisition, Writing – review & editing, Conceptualization, Methodology.
