# Supplementary material for: The evolving landscape and research trend of calcitonin gene-related peptide in migraine: A bibliometric analysis and visualization
Source: Front Neurol. 2024 Jun 24;15:1415760. doi: 10.3389/fneur.2024.1415760 (PMC11228313; doi:10.3389/fneur.2024.1415760)
Supplement: Supplementary file 1 [file Data_Sheet_1.docx]

**Supplementary Figure 1.** The top 15 institutions with the strongest citation bursts.

**
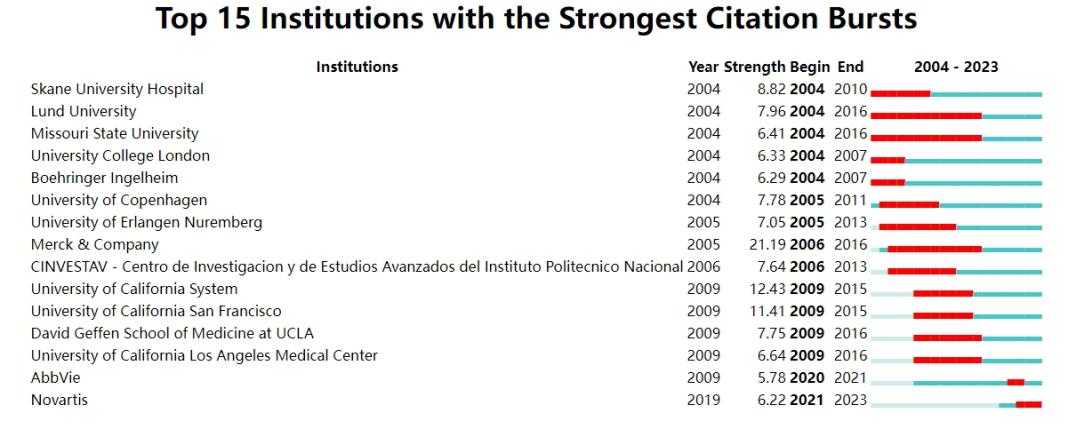
**

**Supplementary Figure 2.** Cooperation maps of authors in CGRP-related research in migraine

**
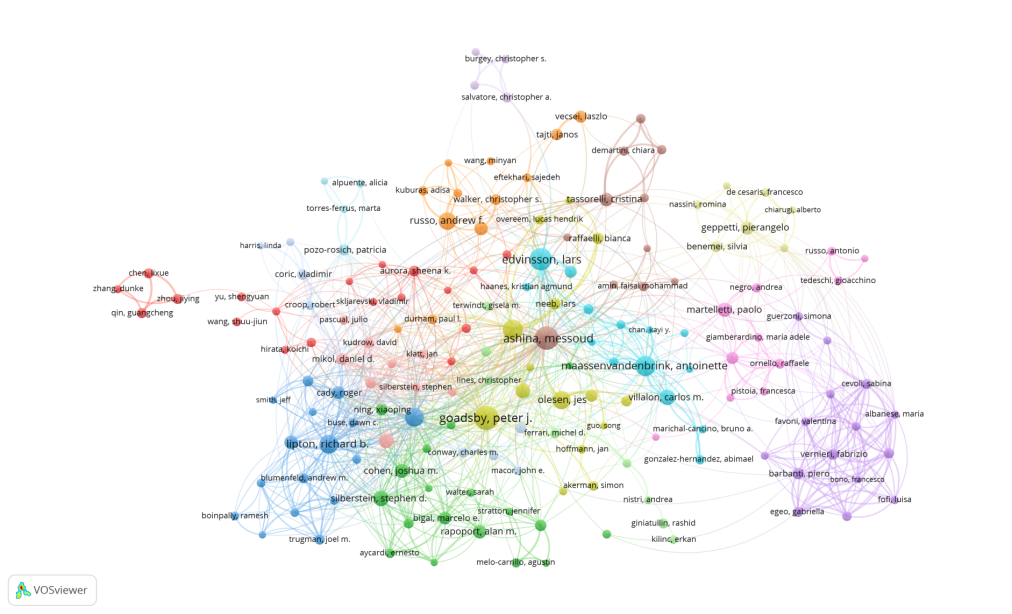
**

**Supplementary Figure 3.** The top 15 keywords with the strongest citation bursts.

**
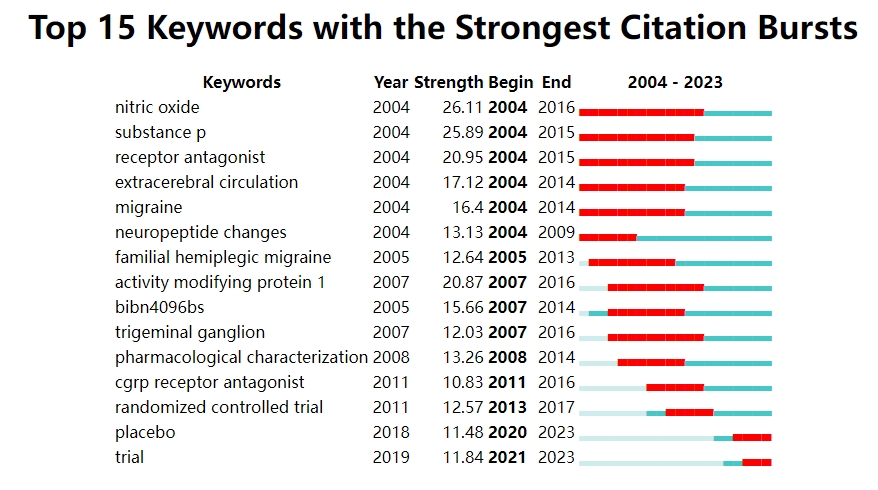
**

**Supplementary Figure 4.** (A) Frequency of keywords plus occurrence over time; (B) Frequency of author keywords occurrence over time on CGRP in migraine. (The size of the circle = term frequency; The gray bar = the first and third quartiles of the occurrence distribution.)

**
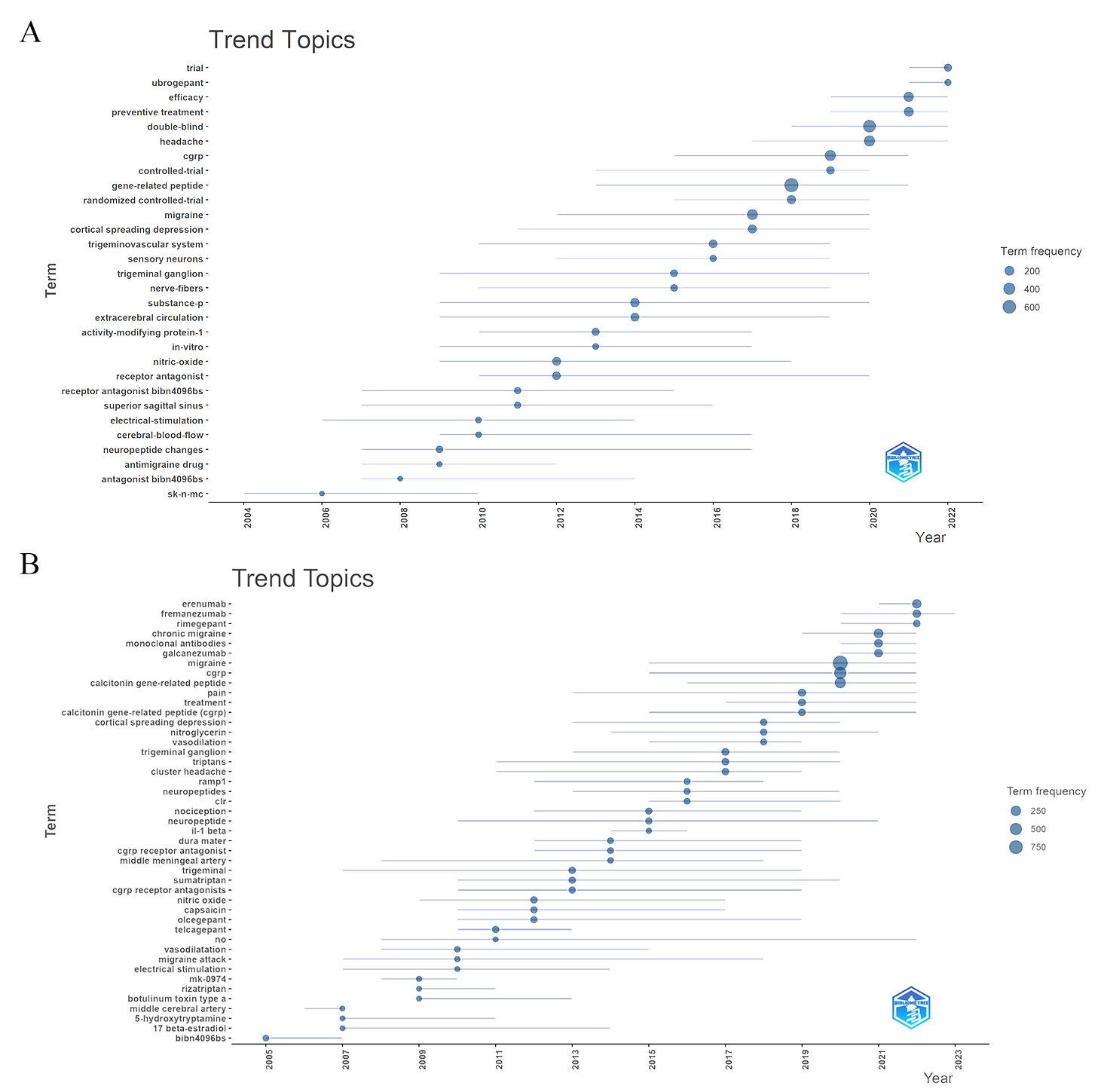
**

**Supplementary Table 1.** The 10 most frequently cited references from 2004 to 2023.

| Rank | **Cited References** | **DOI** | **Local Citations** | **Global Citations** | **LC/GC**  **Ratio (%)** |
| --- | --- | --- | --- | --- | --- |
| 1 | OLESEN J, 2004,  NEW ENGL J MED | 10.1056/NEJMOA030505 | 510 | 927 | 55.02 |
| 2 | OLESEN J, 2018,  CEPHALALGIA | 10.1177/0333102417738202 | 301 | - | - |
| 3 | TEPPER S, 2017,  LANCET NEUROL | 10.1016/S1474-4422(17)30083-2 | 277 | 447 | 61.97 |
| 4 | GOADSBY PJ, 2017,  NEW ENGL J MED | 10.1056/NEJMOA1705848 | 275 | 507 | 54.24 |
| 5 | HO TW, 2008,  LANCET | 10.1016/S0140-6736(08)61626-8 | 261 | 447 | 58.39 |
| 6 | EDVINSSON L, 2018,  NAT REV NEUROL | 10.1038/S41582-018-0003-1 | 244 | 489 | 49.90 |
| 7 | SILBERSTEIN SD, 2017,  NEW ENGL J MED | 10.1056/NEJMOA1709038 | 240 | 427 | 56.21 |
| 8 | HO TW, 2008,  NEUROLOGY | 10.1212/01.WNL.0000286940.29755.61 | 225 | 368 | 61.14 |
| 9 | GOADSBY PJ, 2017,  PHYSIOL REV | 10.1152/PHYSREV.00034.2015 | 218 | 902 | 24.17 |
| 10 | DODICK DW, 2014,  LANCET NEUROL | 10.1016/S1474-4422(14)70128-0 | 217 | 336 | 64.58 |

Abbreviations: DOI, digital object identifier; LC/GC, local citation/global citation.

**Supplementary Table 2.** Top 10 Keywords Plus and Author Keywords.

| Rank | **Keywords Plus** | **Occurrences** | **Author Keywords** | **Occurrences** |
| --- | --- | --- | --- | --- |
| 1 | Gene-related peptide | 697 | Migraine | 987 |
| 2 | Double-blind | 479 | CGRP | 504 |
| 3 | Headache | 301 | Calcitonin gene-related peptide | 333 |
| 4 | CGRP | 292 | Chronic migraine | 165 |
| 5 | Migraine | 279 | Erenumab | 156 |
| 6 | Episodic migraine | 224 | Galcanezumab | 99 |
| 7 | Efficacy | 207 | Monoclonal antibodies | 99 |
| 8 | Preventive treatment | 184 | Fremanezumab | 82 |
| 9 | Safety | 167 | Pain | 65 |
| 10 | Substance-p | 151 | Trigeminal ganglion | 48 |
